# Age-related alterations of angiogenesis, inflammation and bone microarchitecture during fracture healing in mice

**DOI:** 10.1007/s11357-025-01584-y

**Published:** 2025-03-19

**Authors:** Maximilian M. Menger, Ruben Manuschewski, Sandra Hans, Benedikt J. Braun, Moses K. D. El Kayali, Sabrina Ehnert, Emmanuel Ampofo, Selina Wrublewsky, Michael D. Menger, Tina Histing, Matthias W. Laschke

**Affiliations:** 1 Department of Trauma and Reconstructive Surgery, BG Trauma Center Tuebingen, Eberhard Karls University Tuebingen, 72076 Tuebingen, Germany; 2 Institute for Clinical & Experimental Surgery, Saarland University, 66421 Homburg, Germany; 3 Department of Trauma and Reconstructive Surgery, BG Trauma Center Tuebingen, Siegfried Weller Institute for Trauma Research, Eberhard Karls University Tuebingen, 72076 Tuebingen, Germany

**Keywords:** Age, Mice, Fracture healing, Bone healing, Bone remodeling, Angiogenesis, Inflammation

## Abstract

The surgical treatment of geriatric patients represents a major challenge in traumatology. It is well known that aging affects fracture healing. However, the exact pathophysiology of age-related changes in angiogenesis, inflammation and bone remodeling remains still elusive. Therefore, we herein studied the differences of femoral fracture healing in young adult (3–4 months) and aged (16–18 months) CD-1 mice by using a stable closed femoral fracture model with intramedullary screw fixation. The callus tissue was analyzed by means of X-ray, micro-computed tomography (µCT), histology and immunohistochemistry. We found a deteriorated trabecular architecture and a reduced bone formation within the callus tissue of aged mice. Moreover, aged animals showed an increased number of tartrate-resistant acid phosphatase (TRAP)-positive osteoclasts at an early healing time point, whereas the fraction of mature α-smooth muscle actin (SMA)-positive microvessels was significantly reduced. Furthermore, the numbers of macrophages and granulocytes were higher in the callus tissue of aged animals at the end of the healing process. Taken together, these results demonstrate a delayed femoral fracture healing in aged CD-1 mice. This is most likely caused by an early overshooting osteoclast response, a decelerated maturation of the callus microvasculature and a late increased recruitment of pro-inflammatory cells. Targeting these alterations may contribute to the development of novel treatment approaches for the stimulation of bone regeneration in geriatric patients.

## Introduction

The treatment of geriatric patients has become one of the major challenges in trauma and orthopedic surgery. In the year 2020, 53.5 million citizens of the United States of America reached an age of 65 years and older, representing 16% of the overall population [1]. Notably, geriatric patients not only exhibit a higher risk for fractures, but also suffer from an increased morbidity and mortality [2, 3]. Moreover, aging alters bone metabolism by associated degenerative diseases, such as diabetes mellitus, peripheral arterial occlusive disease and osteoporosis, ultimately resulting in a higher rate of delayed healing and non-union formation [4, 5]. However, the exact pathophysiology of age-related changes in angiogenesis, inflammation and bone remodeling during fracture repair still remains poorly understood.

Fracture models in mice represent a powerful tool for preclinical research in traumatology and orthopedics. Among the major advantages of these models are a wide range of gene-targeted strains, short breeding cycles and the availability of monoclonal and polyclonal antibodies. The latter allows the identification of specific cell types and molecules within the callus tissue during bone regeneration and, thus, enables a thorough analysis of angiogenesis, inflammation and tissue remodeling [6]. Holstein et al. [7] established such a preclinical fracture model in mice. They performed a closed femoral fracture and used an intramedullary screw for interfragmentary compression. Notably, the screw provided both rotational and axial stability, therefore providing a reliable fixation of the femoral fracture [7]. Subsequently, this model was used in a plethora of studies for the investigation of fracture healing in mice. These studies covered a wide field of research, including biomechanics [8], impact of comorbidities [9, 10] and the effects of different drugs on bone regeneration [11–13].

In a recent study, we already used the model of Holstein et al. [7] to analyze the effects of aging on fracture healing. In this study, we found an impaired fracture healing in aged mice when compared to young adult animals, indicated by a reduced bending stiffness at 2 and 3 weeks after fracture and a delayed endochondral ossification [14]. However, we did not analyze the age-related changes of angiogenesis, inflammation and bone microarchitecture during the process of fracture repair. Therefore, we performed additional analyses of the obtained specimens in the present follow-up study to investigate in more detail the vascularization, the recruitment of pro-inflammatory cells and the trabecular architecture within the callus tissue of aged animals in comparison to young adult controls. For this purpose, the callus tissue of fractured femora was analyzed by means of X-ray, micro-computed tomography (µCT), histology and immunohistochemistry.

## Material and methods

### Animals and experimental protocol

**Fig. 1:**
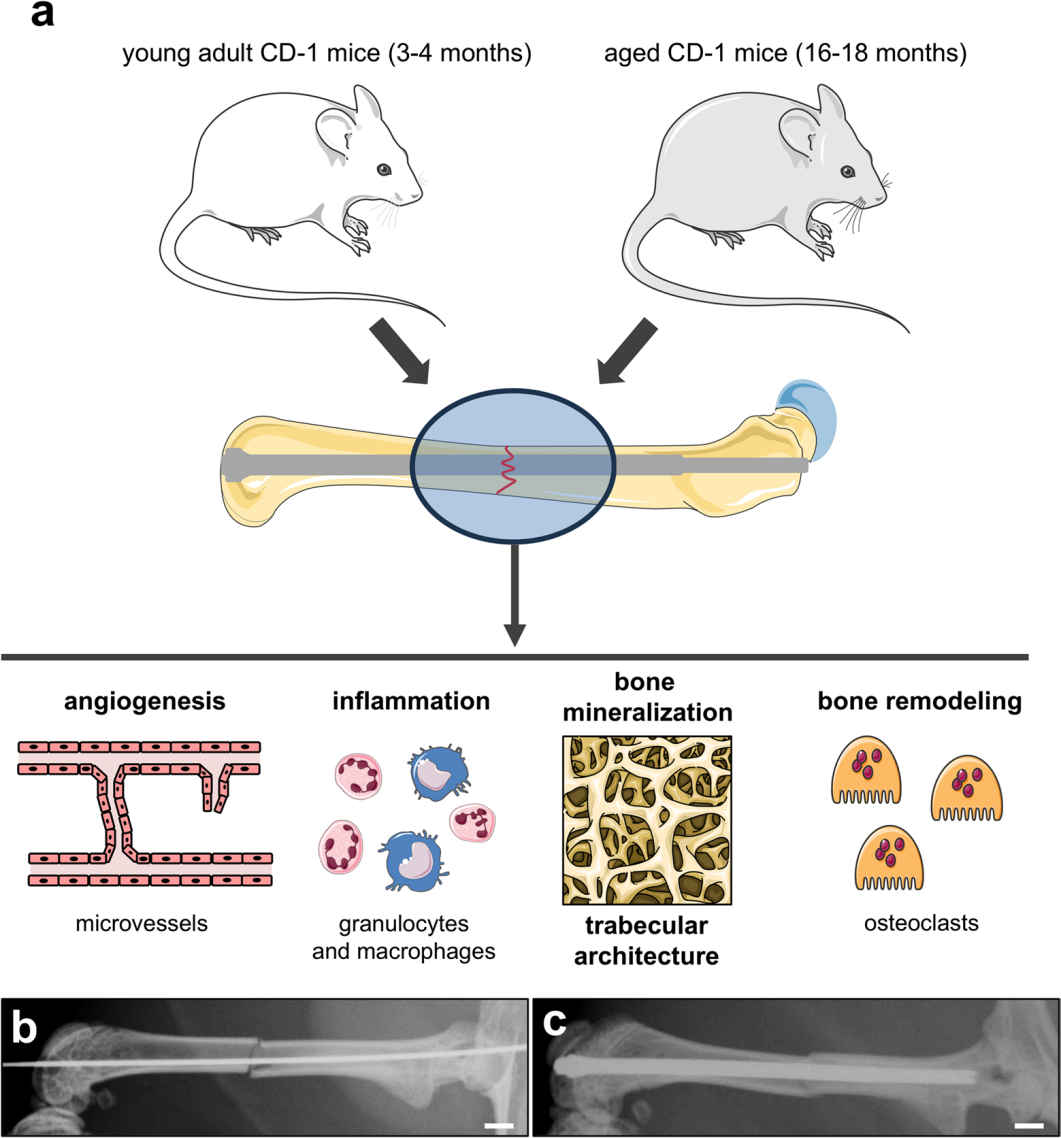
**a:** Experimental setup of the present study. Femoral fracture healing of young adult (3–4 months) and aged (16–18 months) CD-1 mice was analyzed by X-ray, µCT, histology and immunohistochemical analysis. The focus of the investigation included the process of angiogenesis and inflammation at the fracture site as well as the bone mineralization, the trabecular architecture and the bone remodeling within the callus tissue. **b:** X-ray of a fractured mouse femur with an inserted tungsten guidewire during surgery. Scale bar: 1 mm. **c:** Postoperative X-ray after insertion of the intramedullary screw. Scale bar: 1 mm

Specimens from a total number of 100 male and female CD-1 mice with an age of 3–4 months (young adult, n = 50) and 16–18 months (aged, n = 50) were included in the present study. Notably, male and female mice were equally distributed among the study groups to avoid gender as a bias in bone regeneration. In a previous study we have already shown that CD-1 mice with an age of 16–18 months exhibit an altered bone regeneration after fracture [14]. Herein, we now analyzed angiogenesis and inflammation as well as the trabecular architecture and bone remodeling within the callus tissue at 1, 2, 3, 4 and 5 weeks after surgery (young adult and aged mice: n = 10 per group and per time point) (Fig. 1a). The animals were bred at the Institute for Clinical & Experimental Surgery, Saarland University, and kept in groups at a regular light and dark cycle with free access to tap water and standard pellet food (Altromin, Lage, Germany).

### Fracture model

For the investigation of angiogenesis, inflammation and bone remodeling within the callus tissue of young adult and aged mice a closed femoral fracture model with an intramedullary screw (RISystem AG, Landquart, Switzerland) was used [14]. The model was described previously in detail by Holstein et al. [7].

The mice were anesthetized by an intraperitoneal (i.p.) injection of ketamine (75 mg/kg body weight; Ursotamin®, Serumwerke Bernburg, Bernburg, Germany) and xylazine (15 mg/kg body weight; Rompun®, Bayer, Leverkusen, Germany). To guarantee adequate precision, all procedures were performed using an operating microscope. A medial parapatellar incision of ~ 4 mm at the right knee was created and the patella was dislocated laterally. The intramedullary canal was opened by a drill bit (diameter: 0.5 mm) at the intercondylar notch. Then, an injection needle (0.4 mm) was inserted through the greater trochanter over the intramedullary cavity. Afterwards, a tungsten guide wire (diameter: 0.2 mm) was positioned through the needle (Fig. 1b). The femur was fractured by a 3-point bending device, as described previously [15]. Subsequently, the MouseScrew was implanted over the guide wire, providing stabilization of the femoral fracture by interfragmentary compression. The guide wire was removed, the patella was reduced and the wound closure was performed by 5–0 synthetic sutures. Postoperative X-rays confirmed the correct implant position and fracture reduction (MX-20, Faxitron X-ray Corporation, Wheelin, IL, USA) (Fig. 1c). For analgesia the mice received 5 mg/kg body weight carprofen (Rimadyl™, Zoetis GmbH, Berlin, Germany) subcutaneously at the day of surgery. Additionally, tramadol-hydrochloride (Grünenthal, Aachen, Germany) was added to the drinking water (1 mg/mL) one day prior to surgery until three days after surgery.

### X-ray

To guarantee adequate fracture reduction and detect possible fracture or implant dislocation, lateral radiographs were performed before harvesting the femora at the end of the experiments (MX-20, Faxitron X-ray Corporation, Wheelin, IL, USA).

### µCT

The femora were scanned (Skyscan 1172, Bruker, Billerica, MA, USA) at a spatial resolution of 9 μm with a standardized setup (tube voltage: 50 kV; current: 200 μA; intervals: 0.4°; exposure time: 3500 ms; filter: 0.5 mm aluminum). Images were stored in 3-dimensional arrays. Gray values were expressed as mineral content (bone mineral density (BMD)). For this, calcium hydroxyapatite (CaHA) phantom rods with known BMD values (0.250 g and 0.750 g CaHA/cm^3^) were employed for calibration. The region of interest (ROI) defining the novel bone was contoured manually excluding any original cortical bone. The thresholding allowed the differentiation between poorly and highly mineralized bone. The thresholds to distinguish between poorly and highly mineralized bone were based upon visual inspection of the images, qualitative comparison with histological sections and studies investigating bone repair and callus tissue by μCT [16, 17]. A BMD with more than 0.642 g/cm^3^, resulting in gray values of 98–255, was defined as highly mineralized bone. Poorly mineralized bone was assumed to have a BMD value between 0.410 and 0.642 g/cm^3^, resulting in gray values of 68–97.

The following parameters were calculated from the callus ROI for each of the femora: poorly mineralized (pm) bone volume (BV) (mm^3^), highly mineralized (hm) BV (mm^3^), pm BV fraction (pm BV/total tissue volume (TV) (%)), hm BV fraction (hm BV/(TV) (%)), trabecular thickness (mm), trabecular separation (mm), trabecular number (1/mm) and BMD (g hydroxyapatite (HA)/cm^3^).

### Histology

**Figs. 2:**
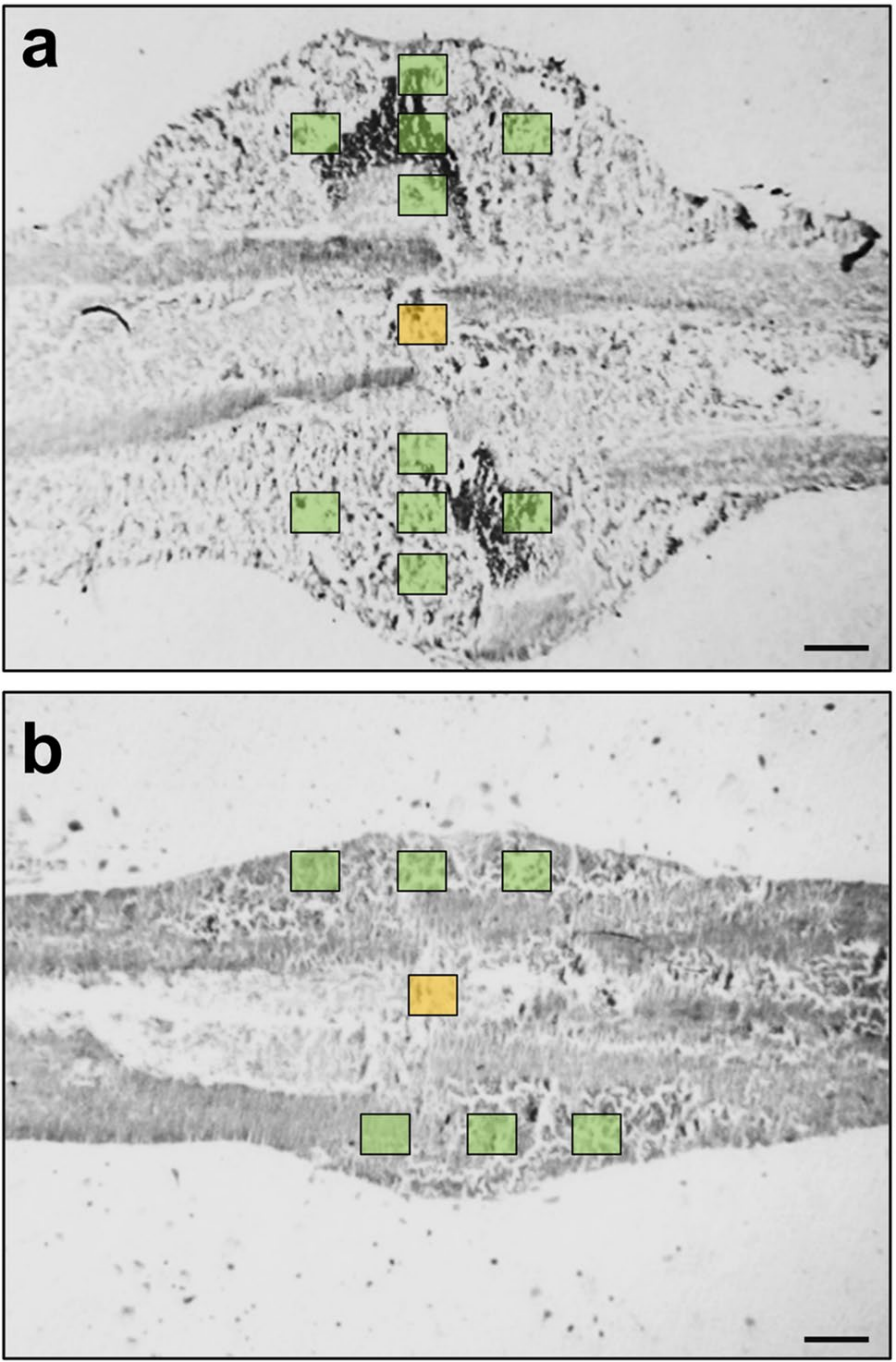
**a and b:** Schematic illustration of the cellular analysis of the callus tissue at 1, 2 and 3 (**a**) as well as 4 and 5 (**b**) weeks after fracture. At 1, 2 and 3 weeks after fracture a single HPF was placed in the central region of the callus (former fracture gap, orange box), while five additional HPFs (green boxes) were placed at each site within the periosteal region of the callus (**a**). At 4 and 5 weeks only 3 additional HPFs were placed at each site within the periosteal region of the callus due to the reduced callus size (**b**). Scale bars: 0.5 mm

After µCT analysis the femora were prepared for histological analyses. Therefore, the bones were fixed in paraformaldehyde for 24 h. Subsequently, the specimens were embedded in a 30% sucrose solution for another 24 h and then frozen at −80 °C. Longitudinal sections through the femoral axis with a thickness of 4 µm were cut by means of the Kawamotos film method [18]. To analyze the process of bone remodeling, tartrate-resistant acid phosphatase (TRAP) activity was analyzed to detect osteoclasts within the callus tissue. For this purpose, longitudinal sections of 4 μm were incubated in a mixture of 5 mg naphthol AS-MX phosphate and 11 mg fast red TR salt in 10 mL 0.2 M sodium acetate buffer (pH 5.0) for 1 h at 37 °C. Sections were counterstained with methyl green and covered with glycerin gelatin. At a 400 × magnification, TRAP-positive cells were counted within the callus tissue in a standardized manner. In 1-, 2- and 3-week specimens, one high-power field (HPF, 400 × magnification) was placed in the central region of the callus (former fracture gap), while five additional HPFs were placed at each site within the periosteal region of the callus. Due to the reduced size of the callus at 4 and 5 weeks, only three additional HPFs were placed at each site within the periosteal region of the callus (Figs. 2a and b). The number of TRAP-positive osteoclasts within each HPF was counted and the mean for each specimen was determined.

### Immunohistochemistry

For the investigation of angiogenesis and inflammation within the callus tissue, additional longitudinal tissue sections were cut for immunohistochemical analyses. For the immunohistochemical detection of microvessels, sections were stained with a monoclonal rat anti-mouse antibody against the endothelial cell marker CD31 (1:100; Abcam, Cambridge, UK). For detecting mature microvessels with a α-smooth muscle actin (SMA) cell layer, the sections were additionally stained with a polyclonal rabbit anti-mouse α-SMA antibody (1:100, Abcam). Cell nuclei were stained with Hoechst 33342 (2 µg/mL; Sigma-Aldrich, Taufkirchen, Germany). In 1-, 2- and 3-week specimens, one HPF (400 × magnification) was placed in a standardized manner in the central region of the callus (former fracture gap), while five additional HPFs were placed at each site within the periosteal region of the callus. Due to the reduced size of the callus at 4 and 5 weeks, only three additional HPFs were placed at each site within the periosteal region of the callus (Figs. 2a and b). The number of CD31-positive endothelial cells and the fraction of mature α-SMA-positive microvessels (%) within each HPF was counted and the mean for each specimen was determined.

For the analysis of the inflammatory response within the callus tissue, the neutrophilic granulocyte marker myeloperoxidase (MPO) and the macrophage marker CD68 were detected. For this purpose, sections were stained with a polyclonal rabbit anti-mouse antibody against MPO (1:100; Abcam) and a polyclonal rabbit anti-mouse antibody against CD68 (1:200; Abcam). The number of MPO-positive granulocytes and CD68-positive macrophages was determined in a standardized manner according to the analysis of microvessels.

### Statistics

All data are given as means ± standard error of the mean (SEM). After proving the assumption for normal distribution (Kolmogorov–Smirnov test) and equal variance (F-test), comparisons between the two experimental groups were performed by an unpaired Student´s t-test. For non-parametrical data, a Mann–Whitney U-test was used. Statistics were performed using the SigmaPlot 13.0 software (Systat Software GmbH, Erkrath, Germany). A p-value < 0.05 was defined to indicate significant differences.

## Results

### Aging affects bone formation and deteriorates the trabecular architecture

**Fig. 3:**
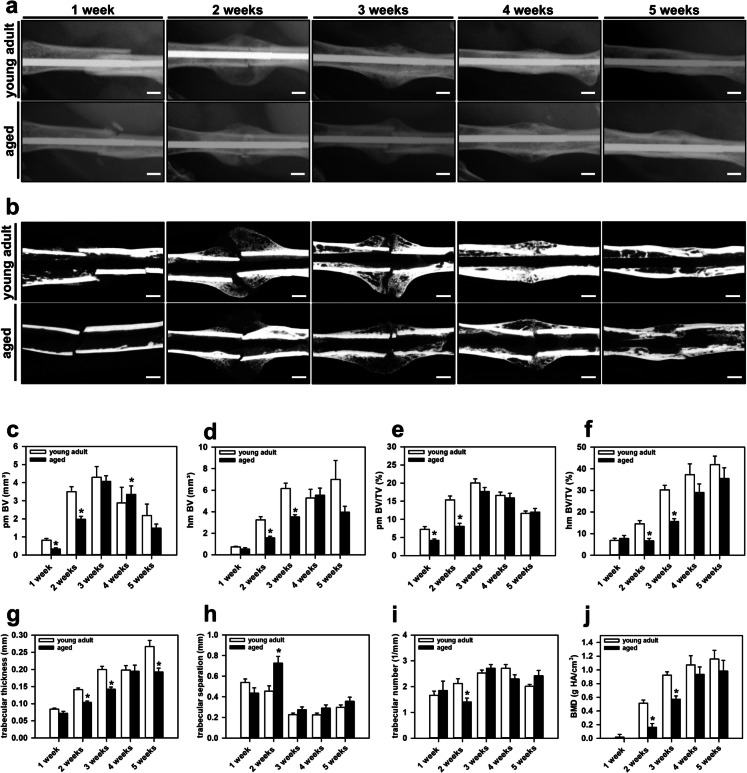
**a:** Representative X-rays of mouse femora in young adult and aged animals at 1, 2, 3, 4 and 5 weeks after fracture. Scale bars: 1 mm. **b:** Representative transversal µCT images of mouse femora in young adult and aged animals at 1, 2, 3, 4 and 5 weeks after fracture. Scale bars: 1 mm. **c-f:** µCT analysis of pm BV (**c**), hm BV (**d**), the ratio of pm BV/TV (**e**) and the ratio of hm BV/TV (**f**) in young adult (*white bars*, n = 10) and aged animals (*black bars*, n = 10). **g-j:** µCT analysis of the trabecular thickness (**g**), trabecular separation (**h**), the trabecular number (**i**) and the BMD (**j**) in young adult (*white bars*, n = 10) and aged animals (*black bars*, n = 10). Mean ± SEM. *p < 0.05 vs. young adult

The X-rays demonstrated endochondral ossification in both study groups with abundant callus formation at 2 and 3 weeks after fracture. During the course of healing, osseous bridging was evident in young adult and aged animals at 4 weeks after fracture. At 5 weeks subsequent bone remodeling occurred, as indicated by a decreasing callus size (Fig. 3a).

In line with the X-ray imaging, the µCT analysis showed typical signs of endochondral ossification at 2 and 3 weeks and osseous bridging at 4 weeks after fracture in both study groups (Fig. 3b). At 1 and 2 weeks after fracture, the pm BV was significantly lower in aged mice when compared to young adult animals. In contrast, at 4 weeks after fracture the, pm BV was significantly higher in aged animals. At 3 and 5 weeks, the pm BV did not significantly differ between the two study groups (Fig. 3c). Furthermore, the hm BV was significantly decreased in aged mice at 2 and 3 weeks after fracture. At 1, 4 and 5 weeks after fracture, however, the analysis revealed no significant differences when compared to young adult animals (Fig. 3d). The ratio of pm BV was significantly lower in aged mice at 1 and 2 weeks when compared to young adult animals, whereas at later study time points the ratio of pm BV did not differ between the two study groups (Fig. 3e). The ratio of hm BV/TV was also significantly lower in aged mice at 2 and 3 weeks after fracture. Of note, at 1, 4 and 5 weeks after fracture, the analysis showed no significant differences between aged and young adult animals (Fig. 3f).

We further investigated the trabecular architecture within the callus tissue. The results revealed a lower trabecular thickness in aged mice at 2, 3 and 5 weeks after fracture, whereas at 1 and 4 weeks no significant differences were found (Fig. 3g). Moreover, the trabecular separation was significantly higher in aged animals at 2 weeks after fracture. Accordingly, the trabecular number was found lower at this time point. The other observation time points showed no significant differences in the trabecular separation and number between the two groups (Figs. 3h, i). Finally, at 2 and 3 weeks after fracture, the BMD within the callus tissue of aged mice was significantly lower when compared to young adult animals. In contrast, at 1, 4 and 5 weeks, the analysis of the BMD showed no significant differences between the two groups (Fig. 3j).

### Aging alters the early and late osteoclastic response

**Fig. 4:**
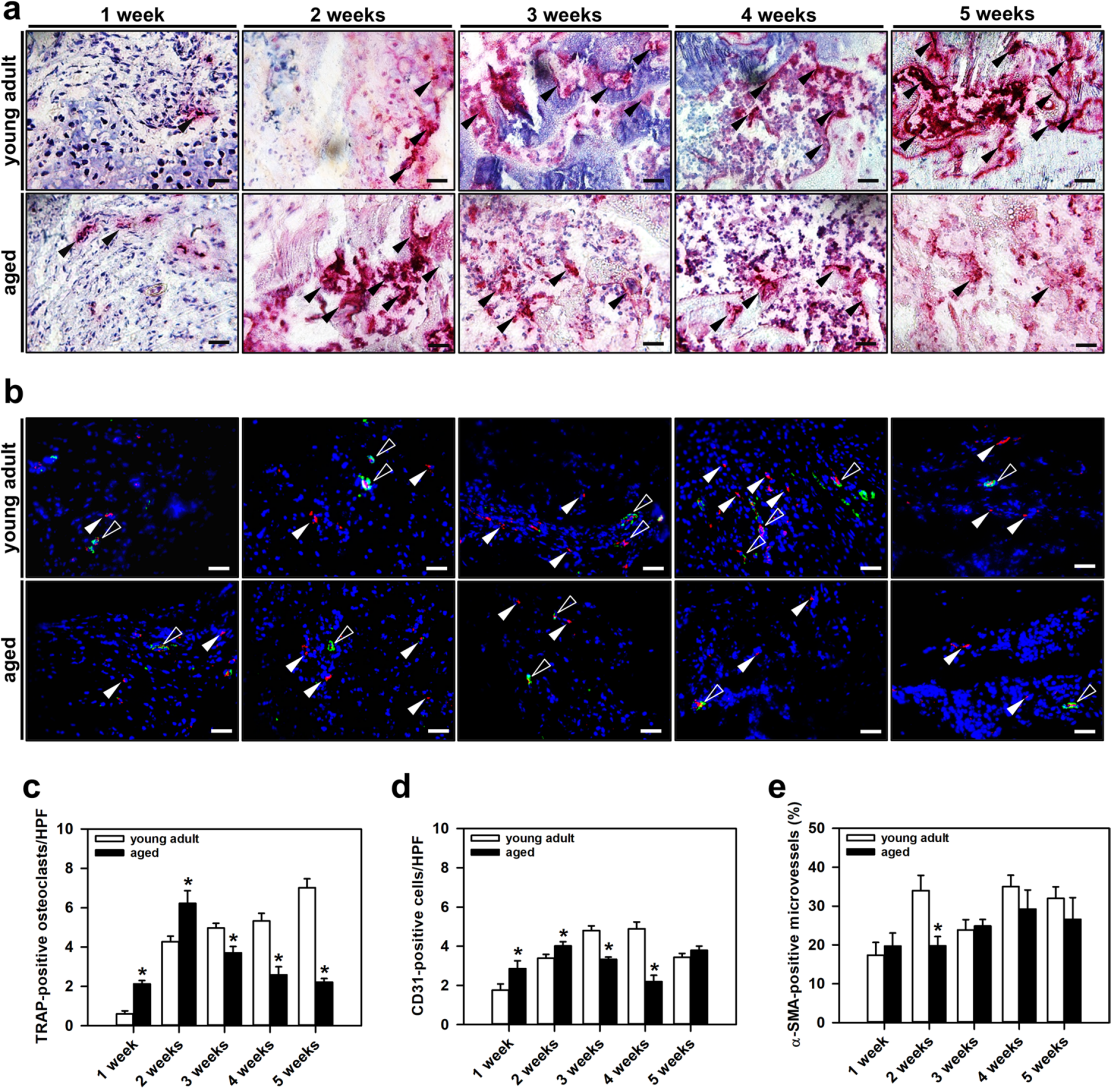
**a:** Representative histological images of TRAP-positive osteoclasts (arrowheads) within the callus tissue of young adult and aged animals at 1, 2, 3, 4 and 5 weeks after fracture. Scale bars: 50 µm. **b:** Representative immunohistochemical images of CD31 and α-SMA staining within the callus tissue of young adult and aged animals at 1, 2, 3, 4 and 5 weeks after fracture. CD31-positive cells (red, white arrowheads) and α-SMA-positive microvessels (green, white hollow arrowheads) are indicated. Scale bars: 50 µm. **c:** Histological analysis of TRAP-positive osteoclasts/HPF in young adult (*white bars*, n = 10) and aged animals (*black bars*, n = 10). **d** and **e:** Immunohistochemical analysis of CD31-positive cells/HPF (**d**) and α-SMA-positive microvessels/HPF (**e**) in young adult (*white bars*, n = 10) and aged animals (*black bars*, n = 10). Mean ± SEM. *p < 0.05 vs. young adult

The process of callus remodeling was analyzed by TRAP-staining of osteoclasts (Fig. 4a). The quantitative analysis revealed a significantly higher number of osteoclasts within the callus tissue of aged mice at 1 and 2 weeks after fracture. In contrast, at 3, 4 and 5 weeks after fracture, the number of osteoclasts was significantly lower in aged mice when compared to young adult animals (Fig. 4a and c).

### Aging delays blood vessel maturation

The process of angiogenesis within the callus tissue was assessed by CD31 and α-SMA staining (Figs. 4b). Interestingly, this staining revealed a higher number of CD31-positive cells at 1 and 2 weeks in aged mice when compared to young adult animals. At 3 and 4 weeks, however, the number of CD31-positive endothelial cells was significantly lower (Figs. 4b and d). Additional analyses of blood vessel maturation demonstrated a lower number of α-SMA-positive microvessels within the callus tissue of aged animals at 2 weeks after fracture (Fig. 4b and e).

### Aging prolongs the inflammatory response

**Fig. 5:**
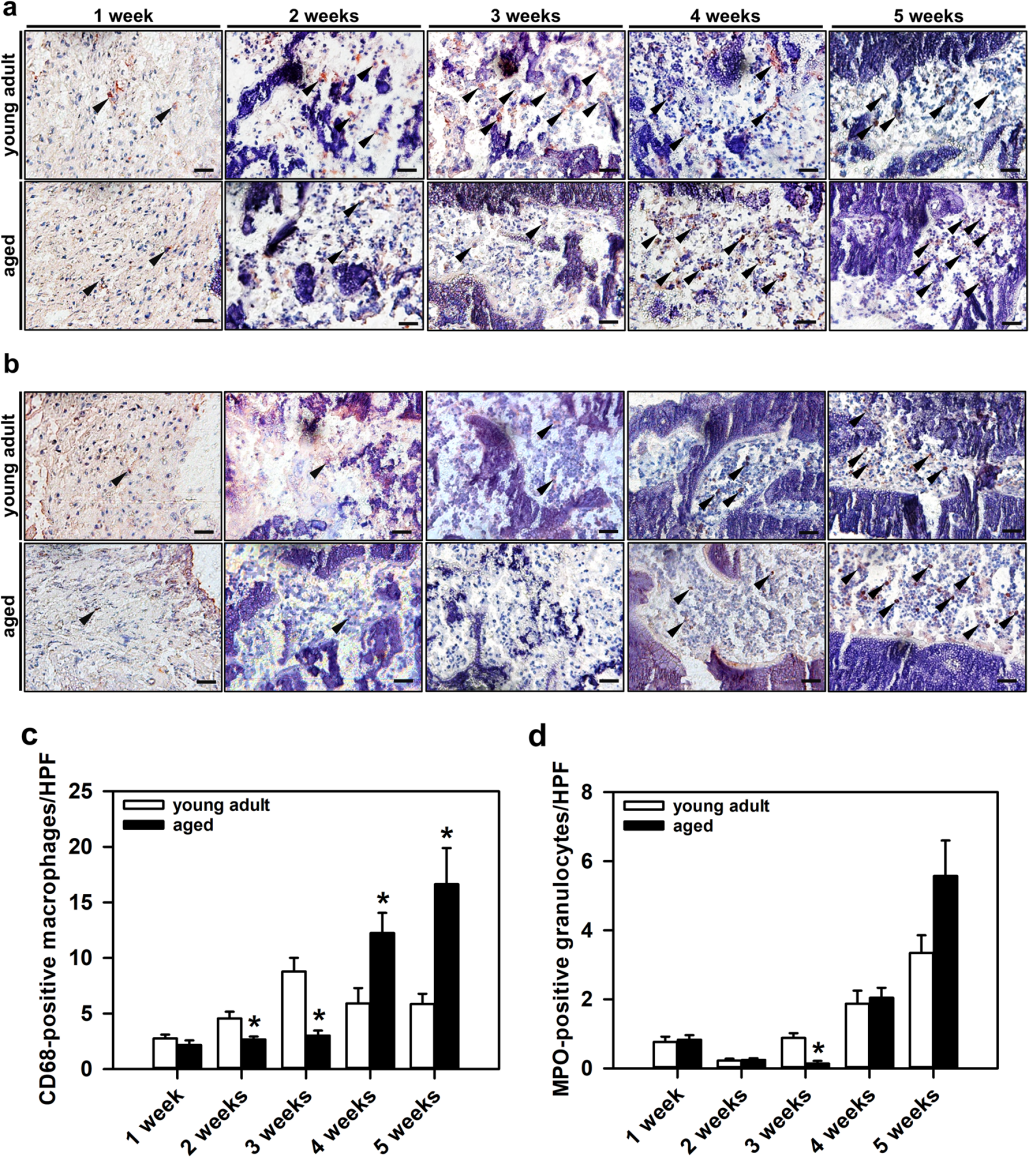
**a:** Representative immunohistochemical images of CD68-positive macrophages (arrowheads) within the callus tissue of young adult and aged animals at 1, 2, 3, 4 and 5 weeks after fracture. Scale bars: 50 µm. **b:** Representative immunohistochemical images of MPO-positive granulocytes (arrowheads) within the callus tissue of young adult and aged animals at 1, 2, 3, 4 and 5 weeks after fracture. Scale bars: 50 µm. **c** and **d:** Immunohistochemical analysis of CD68-positive macrophages/HPF (**c**) and MPO-positive granulocytes/HPF (**d**) in young adult (*white bars*, n = 10) and aged animals (*black bars*, n = 10). Mean ± SEM. *p < 0.05 vs. young adult

The inflammatory response within the callus tissue of aged and young adult mice was analyzed by CD68 staining of macrophages and MPO staining of granulocytes (Figs. 5a and b). In aged mice, the analysis revealed at 2 and 3 weeks a significantly lower number of CD68-positive macrophages, and at 4 and 5 weeks a markedly higher number of macrophages when compared to young adult animals. Notably, at 1 week after fracture, the number of macrophages did not differ between the two study groups (Figs. 5a and c).

The analysis of granulocytes demonstrated a lower number in aged animals at 3 weeks after fracture. The other observation time points showed no significant differences in granulocyte infiltration between the two groups. However, there was a tendency towards a higher number of granulocytes in aged mice at 5 weeks after fracture (Figs. 5b and d).

## Discussion

The present study demonstrates that delayed fracture healing in aged mice is associated with a compromised trabecular architecture, an overshooting early osteoclastic response and a delayed blood vessel maturation of the callus vasculature in the early healing phase. Furthermore, the fracture site in aged animals is characterized by an elevated and prolonged inflammatory response, as indicated by an increased recruitment of macrophages within the callus tissue in the late healing phase.

Murine fracture healing models are of increasing interest in preclinical trauma and orthopedic research [6]. However, the development of a reliable, standardized and reproducible fracture healing model in mice represents a major challenge due to the small size of the animals. Hence, in some studies, the fracture was left unstabilized [19, 20]. This may not be recommended because an unstabilized fracture does not mimic the stable osteosynthesis performed in patients, which makes the clinical translation of the obtained results difficult. Moreover, an unstabilized fracture causes additional loss of function and pain for the animal, which contradicts animal welfare standards. Accordingly, a plethora of different techniques for fracture fixation in mice have been developed throughout the last decades, including external fixators [21], plates [22], nails [23] and screws [7]. In the present study, we used a closed femoral fracture model, which achieved fracture stabilization by an intramedullary screw. Notably, the screw allows interfragmentary compression by a cone-shaped head and a proximal thread, thereby guaranteeing both rotational and axial stability [7]. Moreover, the fracture model has the distinct advantage of providing a trauma-induced fracture, instead of an artificially created osteotomy. Hence, the present model may mimic fracture healing in clinical practice as closely as possible.

There is increasing evidence that aging and aging-associated osteoporosis have detrimental effects on fracture repair. In fact, a high incidence of implant fixation failure in aged patients as well as an increased rate of delayed healing and non-union formation indicate a reduced healing capacity [4, 5, 24]. Most preclinical studies use rodent osteoporosis models for investigating the effects of aging on fracture repair. For instance, Wang et al. [25] showed a delayed bone formation and impaired biomechanical properties in ovariectomy-induced osteoporosis in rats. Moreover, Namkung-Matthai et al. [26] demonstrated a reduced callus size and BMD in osteoporotic rats at 3 weeks after fracture. However, several other studies reported that osteoporosis has no significant effect on bone regeneration. In fact, Langeland et al. [27] revealed that the collagen content and tensile strength of tibial fractures do not differ between ovariectomized rats and corresponding control animals. Melhus et al. [28] confirmed these findings by demonstrating that osteoporosis and vitamin D deficiency do not significantly affect bone regeneration in rats. When investigating age-related effects on fracture repair, it should be considered that there are several other factors than osteoporosis with a potential impact on bone regeneration. These include, for instance, age-related changes in stem cell differentiation and inflammation [29]. These factors are not present in models of ovariectomy-induced osteoporosis. Therefore, we used aged CD-1 wildtype mice to accurately mimic the process of aging in geriatric patients.

The µCT analyses of the present study demonstrated a delayed fracture healing, as indicated by a decreased pm and hm BV at early healing time points in aged mice. However, the analysis showed no significant difference in BV at 5 weeks after fracture when compared to young adult animals. Thus, the overall bone healing capacity of geriatric animals seems not to be affected by aging. These findings are in line with our previous study, which investigated the biomechanical characteristics of fracture repair in young adult and aged mice [14]. In addition, Lopas et al. [30] demonstrated that geriatric C57BL/6 mice preserve their ability for endochondral and intramembranous ossification.

The µCT analyses additionally revealed distinct changes in the trabecular architecture between young adult and aged CD-1 mice, particularly at 2 weeks after fracture. In fact, we found a significantly reduced trabecular thickness and number in aged animals. Accordingly, the trabecular separation was increased when compared to young adult mice. These findings were associated with an increased number of TRAP-positive osteoclasts within the callus tissue. Of note, osteoclast-mediated bone resorption is essential for successful bone regeneration and subsequent bone remodeling, eventually resulting in laying down mature lamellar bone [31]. However, while this takes place at later time point of healing, an overshooting osteoclast-mediated bone resorption at an early healing time point may more probably impair the process of bone regeneration by reducing the amount of novel bone tissue. In line with this view, we demonstrated in a previous study that pantoprazole treatment impairs fracture repair in aged CD-1 mice most likely by an early, overwhelming osteoclastic response [32]. The increased osteoclastic activity at an early healing time point may be caused by a higher interferon (IFN)-γ activity in aged animals, which in turn stimulates the expression of the receptor activator of NF-κB ligand (RANKL) [33, 34]. Notably, RANKL is a potent stimulator of osteoclastogenesis by binding RANK on the osteoclast cell membrane [35, 36]. Of interest, at 3 to 5 weeks after fracture, the aged animals showed a significantly lower number of osteoclasts compared to young adult controls. This reduced osteoclastic activity at later time points of the healing process may explain the slightly increased callus diameter when compared to the young adult animals [14], indicating a delay of bone remodeling.

Vascularization is a major prerequisite for successful fracture repair. Newly formed microvessels supply the callus tissue with vital nutrients, oxygen and osteogenic progenitor cells. Accordingly, disturbance of the vascularization at the fracture site may markedly impair bone healing. For instance, pharmacological inhibition of angiogenesis by TNP-470 and non-steroidal anti-inflammatory drugs (NSAIDs) has been shown to suppress fracture repair and to promote non-union formation [37, 38]. Notably, the skeleton of aged individuals is associated with a dysfunction of the bone vascular system [39]. The vasculature of the skeleton in aged rats exhibits a reduced perfusion due to a decreased patency of blood vessels within the bone marrow when compared to young adult animals [40]. This deteriorated vascularization may decrease the process of angiogenesis at the fracture site. In fact, Lu et al. [41] found not only a lower amount of blood vessels within the callus tissue at an early healing time point, but also a delayed expression of the pro-angiogenic factors vascular endothelial growth factor (VEGF), hypoxia inducible factor (HIF)−1α and matrix metalloproteinase (MMP)−9 and −13 in middle-aged and aged mice when compared to juvenile mice [41]. In contrast to these findings, our immunohistochemical analysis demonstrated a higher number of CD31-positive cells within the callus tissue of aged animals at 1 and 2 weeks after fracture. However, the sole detection of endothelial cells does not necessarily prove a functional vascular network. Similar to vascular structures in non-unions, the early increased blood vessel sprouting observed in aged mice may be due to hypoxia within the callus tissue [42, 43]. In line with this hypothesis, α-SMA staining confirmed a lower percentage of mature blood vessels at 2 weeks after fracture in aged mice when compared to young adult animals. Hence, the delayed fracture healing observed in aged mice may be due to an impaired formation of mature, functional blood vessels during the early healing process.

An adequate inflammatory response is of major importance to initiate the process of bone regeneration. Granulocytes and macrophages are among the first immune cell types infiltrating the fracture site and stimulating the migration of osteogenic precursor cells within the callus tissue [44]. Notably, the resolution of inflammation is also of pivotal importance for successful fracture repair. Aging, however, is associated with a chronic, low-grade systemic inflammation, which is characterized by a systemic elevation of pro-inflammatory cytokines and monocytes, also referred to as “inflammaging” [45–47]. Interestingly, this chronic inflammatory state in the aged deteriorates the process of fracture healing most likely by an increased osteoclast activation and decreased osteoblast formation. Moreover, there is evidence that a chronically elevated inflammation decreases the number and function of skeletal stem cells [44, 47]. In line with these findings, we found an increased number of macrophages and granulocytes at the end of the bone healing process in aged mice when compared to young adult mice, indicating a persisting inflammatory response. Surprisingly, the analysis demonstrated a lower number of pro-inflammatory cells at 2 and 3 weeks after fracture in aged mice. This reduced inflammation at an early healing time point may have contributed to the observed delay in bone healing in aged animals. The exact reason for the decreased inflammatory response at the beginning of fracture repair has yet to be determined. One possible explanation may be the delayed maturation of the callus tissue vasculature in aged mice, thus, limiting the recruitment of pro-inflammatory cells at the fracture site.

In summary, the present study delineates age-related changes in angiogenesis, inflammation and bone remodeling during femoral fracture healing in mice. We found a deteriorated trabecular architecture, an early overshooting osteoclastic response and a delay in blood vessel maturation in aged animals. Moreover, the callus tissue of aged mice exhibited an increased and persisting infiltration of inflammatory cells at the end of the fracture repair process. These pathophysiological alterations may explain the delayed bone healing observed in the aged. Hence, specifically targeting these alterations may contribute to the development of novel treatment approaches for the stimulation of bone regeneration in geriatric patients.

## Data Availability

The data generated and analyzed during the current study are available from the corresponding author upon reasonable request.
